# Atrazine adsorption removal with nylon6/polypyrrole core-shell nanofibers mat: possible mechanism and characteristics

**DOI:** 10.1186/s11671-015-0903-6

**Published:** 2015-05-06

**Authors:** Bi-Yi Yang, Yang Cao, Fei-Fei Qi, Xiao-Qing Li, Qian Xu

**Affiliations:** Key Laboratory of Environmental Medicine Engineering, Ministry of Education, School of Public Health, Southeast University,87 Dingjiaqiao, Nanjing, 210009 China; Suzhou Key Laboratory of Environment and Biosafety, Suzhou, 215123 China

**Keywords:** Polypyrrole, Nanofibers, Atrazine, Adsorption mechanism, Adsorption characteristics, Removal

## Abstract

A functionalized nylon6/polypyrrole core-shell nanofibers mat (PA6/PPy NFM) was prepared *via situ* polymerization on nylon6 electrospun nanofibers mat (PA6 NFM) template and used as an adsorbent to remove atrazine from aqueous solutions. The core-shell structure of PA6/PPy NFM can be clearly proved under scanning electron microscope (SEM), transmission electron microscopy (TEM), and X-ray photoelectron spectroscopy (XPS). The effects of initial solution pH and ionic strength, as well as the comparison of the adsorption capacity of functionalized (PA6/PPy NFM) and non-functionalized (PA6 NFM) adsorbent, were examined to reveal the possible adsorption mechanism. The results indicated that π-π interaction and electrostatic interaction should play a key role in the adsorption process. The kinetics and thermodynamics studies also further elucidated the detailed adsorption characteristics of atrazine removal by PA6/PPy NFM. The adsorption of atrazine could be well described by the pseudo-second-order equation. The adsorption equilibrium data was well fitted with the Freundlich isotherm model with a maximum adsorption capacity value of 14.8 mg/g. In addition, the increase of adsorption rate caused by a temperature increase could be felicitously explained by the endothermic reaction. The desorption results showed that the adsorption capacity remained almost unchanged after six adsorption/desorption cycles. These results suggest that PA6/PPy NFM could be employed as an efficient adsorbent for removing atrazine from contaminated water sources.

## Background

Atrazine (2-chloro-4-ethylamine-6-isopropylamino-1,3,5 triazine) is one of the most popular herbicides used in agriculture and forestry industries [1-3]. Due to its widespread application and persistence, a long time, even decades, is required for atrazine to degrade in water and soil. Moreover, atrazine were recently considered as a potential carcinogen by many of reports [4-6]. The consumption of contaminated water is a major route of human exposure to the pollutant [7]. Accordingly, many countries have set the residual level for atrazine in ground water and drinking water. The standard of European Union for the residues of atrazine herbicides is limited to 0.1 μg/L for single herbicides including atrazine and 0.5 μg/L for the sum of the herbicides in drinking water [8]. According to US environmental protection agency (USEPA), the maximum contaminant level (MCL) is defined at 0.003 mg/L for drinking water [9]. Similarly, the State Environmental Protection Administration of China has set the MCL of atrazine as 0.003 mg/L in the centralized water supply from surface water (Environmental Quality Standard for surface Water of China, GB 3838–2002). Hence, it is urgent to develop an efficient treatment technique to remove atrazine from water environment.

Atrazine can be hardly removed by routine treatment technique, such as coagulation and filtration [10]. Therefore, it is necessary to develop some treatment methods which are more efficient. Adsorption has evolved into one of the most frequently used physical processes for atrazine removal due to its simplicity and easy operation [11]. The key of the adsorption process is the choice of absorbing material. The search for new adsorbents with higher removal efficiency seems to be a never-ending task for practical applications.

In recent years, nanofibers, which can be easily fabricated by a process commonly known as electrospinning (e-spinning), have attracted great interest from the field of sorbent material for solid phase extraction (SPE) due to their remarkably high sorptive capacity [12,13]. Our related research, using PA6 NFM as SPE sorbent, has demonstrated high effectiveness in extracting some non-polar and medium polar compounds from aqueous samples [12-17]. Compared with microscale adsorbents, electrospun nanofibers with a higher surface-to-volume ratio can achieve larger specific surface and generate more active sites for adsorption. Therefore, the adsorption of the target compounds is facilitated and achieved with a small amount of nanofiber (2 ~ 3 mg). Furthermore, some researchers have indicated that using polymer fiber mat as the adsorbent can avoid the subsequent separation process [18]. Due to the facts mentioned above, the electrospun nanofiber mat is considered to have great potential as an efficient adsorbent in hazardous materials removal. Indeed, polymer nanofibers obtained by electrospinning have shown excellent removal ability for heavy metal ions and anionic dyes in water [18-25]. However, limited by the simple chemical structure of electrospun nanofibers, they were hardly used to remove organic pollutants, e.g., atrazine. So there is new demand to find the proper material to fabricate nanofibers that can be used as the ‘right’ adsorbent.

According to our preliminary study, atrazine had low adsorption capacity to the PA6 nanofibers due to its higher polarity. Thus, our goal is to develop functionalized nanofibers, which can enhance the adsorption ability while maintaining the advantages of nanofibers. Recent research shows that polypyrrole (PPy) has a high extraction efficiency for polar adsorbates because multiple types of interactions with the target compounds are involved, such as the π-π interactions, hydrogen bonding, acid–base property, ion-exchange properties, and electrostatic attractions [26-28]. As a result, polar target compounds might be adsorbed through more than one type of interactions. The mixed-mode interactions can not only make the adsorption of polar target compounds possible but also enhance the adsorption capacity. In this respect, PPy nanofibers can guarantee a high efficiency in atrazine removal from contaminated water because the multifunctional properties of PPy will be combined with high adsorption capacity of electrospun nanofibers.

However, PPy nanofibers are hard to be fabricated directly due to its insolubility and infusibility. In this study, PA6/PPy NFM was prepared *via situ* polymerization on PA6 NFM template and used as an adsorbent for atrazine in aqueous solutions. As the key issues aroused in the feasibility of atrazine removal with PA6/PPy NFM in wastewater treatment, the possible adsorption mechanism, kinetic, and thermodynamic characteristics were investigated.

## Methods

### Materials

High-purity atrazine standard was purchased from Shanghai Pesticide Research Institute (Xuhui, Shanghai, China). High-performance liquid chromatography (HPLC)-grade methanol, acetonitrile, and acetone used in analysis were obtained from Tedia Inc, Fairfield, OH, USA. Analytical reagent grade cresol, formic acid, hydrochloric acid, pyrrole monomer, and sodium hydroxide were purchased from Chemical Reagent Factory, Shanghai, China. Nylon6 was purchased from Nanjing DebioChem Co, Ltd, Nanjing, China.

### Preparation of PA6/PPy NFM

PA6 NFM was fabricated into a template by electrospinning as previous description [12,15,29]. PPy was then coated on PA6 by *situ* polymerization with FeCl_3_ as an oxidant and Cl^−^ as a dopant. The procedure was briefly described as follows. An appropriate amount of PA6 was dissolved in a composite solvent of formic acid and m-cresol (6:4, *v*/*v*). The PA6 nanofibers were spun at 15 kV with a 7-gauge needle at a 0.1 mL/h feeding rate. A piece of aluminum foil was used to collect random PA6 NFM mat for 2 h.

The PA6 NFM was cut into pieces of 10 cm × 10 cm size then immersed in 50 mL anhydrous ethanol solution of pyrrole monomer (0.1 mol/L) for 1 h. The polymerization of pyrrole monomer was initiated by the addition of same volume of anhydrous ethanol solution of FeCl_3_ (0.23 mol/L). The *situ* polymerization lasted for 24 h at room temperature. The PA6 NFM changed appearance from white to black as a result of the coating of the black PPy during the polymerization. The synthesized PA6/PPy NFM was washed with anhydrous ethanol, followed by water for several cycles until the solution turned colorless, and then air-dried. For thorough examination of the core-shell structure, the PA6/PPy NFM was soaked in formic acid for 12 h to remove the cores, followed by several water washes and final vacuum drying prior to the test.

### Characterization

The morphological images of the PA6 NFM and PA6/PPy NFM were obtained using a SEM (Hitachi S-3000 N, Japan), at an acceleration voltage of 5 kV. A TEM (JEOL-2100, Akishima-shi, Tokyo, Japan) system was employed to examine the core-shell structure of the PA6/PPy NFM. The surface-to-volume ratio of NFM was measured with the ASAP 2020 accelerated surface area and porosimetry system (Micromeritics Instrument Corporation, Norcross (Atlanta), GA, USA). Fourier transform infrared (FTIR) spectra were carried out on a FTIR spectrophotometer (NEXUS 870, ThermoNicolet; ThermoFisher Scientific, Waltham, MA, USA). The surface chemical compositions of PA6 nanofibers and PA6/PPy NFM were detected with XPS (ESCALAB 250Xi, ThermoFisher Scientific, Waltham, MA, USA).

### Quantitative method for atrazine

Quantitative analysis of atrazine was performed using high-performance liquid chromatography (HPLC, Shimadzu, Japan) including a LC-10A HPLC pump. The analysis was completed with the following conditions: Diamonsil C18 reverse-phase column (5 μm, 250 × 4.6 mm) and a solution of acetonitrile:water (4:1) as the mobile phase at a flow rate of 1.0 mL/min using isocratic elution with UV detection at 220 nm.

### Adsorption mechanism experiments

To investigate the adsorption mechanism on atrazine removal, the effect of solution pH and ionic strength was studied. A 2.0-mg PA6/PPy NFM was immersed into 2 mL of 10 mg/L atrazine aqueous solution in a 10-mL glass conical flask with cover for 12 h at 293 K.

The effect of pH value on the adsorption of atrazine using PA6/PPy NFM was tested by varying solution pH from 3 to 11. The pH of the solution was adjusted using 0.1 M HCl or 0.1 M NaOH. Meanwhile, to study the effect of ionic strength on the adsorption of atrazine, a series of experiments was carried out with the concentrations of NaCl changed from 2.5% to 10% and the pH of the initial solution remained at 7.

The adsorption capacities of PA6 NFM and PA6/PPy NFM were also tested for comparison purpose under the same experimental conditions. A 2.0-mg PA6/PPy NFM was added into 2 mL of atrazine solutions at two initial concentrations of 1 and 10 mg/L. The adsorption experiment was carried out at 293 K and continued for 12 h to reach adsorption equilibrium.

### Adsorption experiments

The adsorption of atrazine from aqueous solutions was examined by batch adsorption experiments. A 2.0-mg PA6/PPy NFM was immersed into 2 mL aqueous solution at a desired concentration in a 10 mL glass conical flask with cover at different temperatures for 12 h to reach adsorption equilibrium. HPLC-UV method described at the ‘Quantitative method for atrazine’ section was utilized to quantitate the concentrations of adsorbate.

The adsorption kinetics was studied with 2.0 mg PA6/PPy NFM in 10 mg/L atrazine solutions incubated at 293, 303, 323, and 343 K, respectively. Samples of 50 μL withdrawn from the solutions in the course of adsorption were collected at time intervals of 5, 10, 15, and 30 min, 1, 2, 3, 4, 5, 6, 7, 8, 10, and 12 h for atrazine analysis using the quantitative method described in ‘Quantitative method for atrazine’ section. The same volume of blank solvent was added at each scheduled time intervals. The adsorption capacity can be calculated by following equation [18]:

1 $$ {q}_t=\frac{C_0\hbox{-} {C}_t}{m}\times V $$

where *q*_*t*_ is the adsorption capacity at time *t* (mg/g), *C*_0_ is the initial concentration of atrazine in solution (mg/L), *C*_*t*_ is the atrazine concentration at time *t* (mg/L), *m* is the mass of adsorbent (g), and *V* is the volume of solution (L).

All the adsorption isotherm experiments were carried out at a serial atrazine concentrations of 1 2, 4, 5, and 10 mg/L at the temperatures of 293, 323, and 343 K. A 50-μL sample was withdrawn from the solutions after the equilibrium was reached. The equilibrium adsorption capacity was calculated by the following equation [18]:

2 $$ {q}_e=\frac{C_{\mathrm{o}}\hbox{-} {C}_{\mathrm{e}}}{m}\times V $$

where, *q*_e_ is the equilibrium adsorption capacity (mg/g), *C*_0_ is the initial concentration of atrazine in solution (mg/L), *C*_e_ is the equilibrium concentration (mg/L), *m* is the mass of adsorbent (g), and *V* is the volume of solution (L).

For the study of the maximum adsorption capacity, 2.0 mg PA6/PPy NFM was used to adsorb atrazine at different initial concentrations (0 to 200 mg/L) at 293 K for 12 h until equilibrium was reached.

Independent blank experiments found that there was no atrazine adsorption to the glass conical flasks. All the experiments mentioned above were performed in triplicates.

### Reusability of PA6/PPy NFM

In order to evaluate the reusability, 2.0 mg PA6/PPy NFM was first contacted with atrazine in 2 mL of 10 mg/L solution for 12 h at 298 K. After adsorption, PA6/PPy NFM was soaked in 2 mL methanol followed by 2 mL distilled water for 5 min respectively to elute the adsorbed atrazine. Before the next adsorption, PA6/PPy NFM was air-dried at room temperature. The procedure above was repeated for six times to test the reusability of the adsorbent.

## Results and discussion

### Characterization of the PA6/PPy NFM

#### Morphology of the PA6/PPy nanofibers

**Figure 1:**
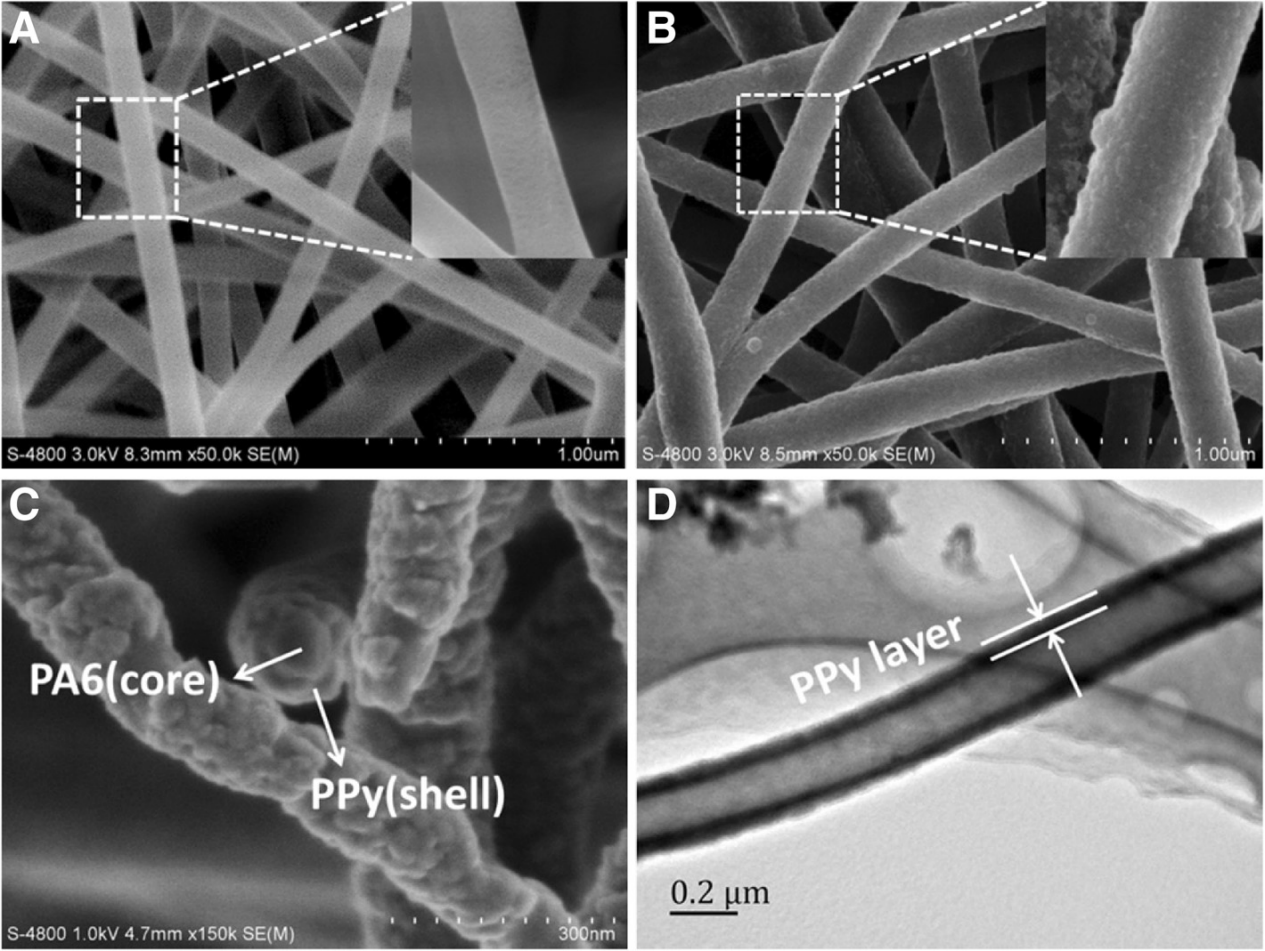
SEM images of PA6 nanofibers **(A)**, PA6/PPy nanofibers **(B,C)**, and TEM image of PPy hollow fibers **(D)**. The morphological images of the PA6 ENF and PA6/PPy NFM were obtained with a scanning electron microscope (SEM; Hitachi S-3000 N). A transmission electron microscopy (TEM) system was employed to examine the core-shell structure of the PA6/PPy NFM. PA6 nanofibers had smooth surface and the average diameter was about 200 nm. While for PA6/PPy nanofibers, the average diameter increased to 220 nm, and the surface became coarse. The cross-section of PA6/PPy nanofibers and TEM image indicated that the PA6 610 nanofibers were coated with the PPy, forming a core-shell structure.

The morphology of PA6 NFM and PA6/PPy NFM was studied under SEM and TEM (Figure 1). PA6 nanofibers had smooth surface, and the average diameter was about 200 nm (Figure 1A). After the pyrrole polymerization, the average diameter was increased to 220 nm, and the surface of the PA6/PPy nanofibers became coarse (Figure 1B). The cross-section of PA6/PPy nanofibers (Figure 1C) indicated that the PA6 nanofiber was coated with the PPy, forming a core-shell structure. The evenly and continuously spread PPy layer also proved that PPy was successfully coated on the PA6 nanofibers to form PA6/PPy nanofibers. After treated with formic acid for 12 h, the PPy hollow fibers were obtained as the PA6 nanofibers template was dissolved, leaving PPy layer intact. The core-shell structure of PA6/PPy nanofibers was further confirmed with the TEM images of PPy hollow fibers (Figure 1D). It demonstrates that the functionalized nanofibers can be formed by employing PA6 as core and PPy as shell.

#### ATR-FTIR characteristic of the PA6/PPy NFM

**Figure 2:**
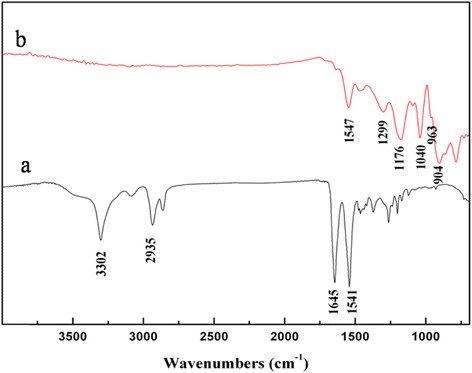
ATR-FTIR spectra of PA6 NFM **(a)** and PA6/PPy NFM **(b)**. Fourier transform infrared (FTIR) spectra were carried out on a FTIR spectrophotometer (NEXUS 870). After polymerization of pyrrole on the nanofibers, the characteristic vibrational bands of PPy are observed to grow. When comparing with PA6 nanofibers, the characteristic IR peaks of PA6 disappeared in the FTIR spectrum.

The results of ATR-FTIR were shown in Figure 2. After polymerization of pyrrole on the nanofibers, the characteristic vibrational bands of PPy are observed to grow. These peaks are pyrrole ring stretching at 1,547 cm^−1^, conjugated C-N stretching at 1,176 cm^−1^, C-H stretching vibration at 963 cm^−1^, and C-H deformation at 904 cm^−1^, respectively. These peak positions are well matched with the reported peaks for PPy [30]. However, compared with PA6 nanofibers, the characteristic IR peaks of PA6 disappeared in the FTIR spectrum, such as the N-H and C-H stretching vibrations at 2,800 to 3,300 cm^−1^ and the coupled motions of C = O stretching and N-H in-plane bending at 1,500 to 1,700 cm^−1^ [31]. In addition, the peaks at 1,299 and 1,040 cm^−1^ were assigned to the in-plane vibrations of C-H and in-plane bending mode. The spectrum of PA6/PPy nanofibers clearly exhibits the characteristic peaks with respect to PPy, which indicates the formation of PPy in nanofibers.

#### XPS analysis of the PA6/PPy NFM

**Figure 3:**
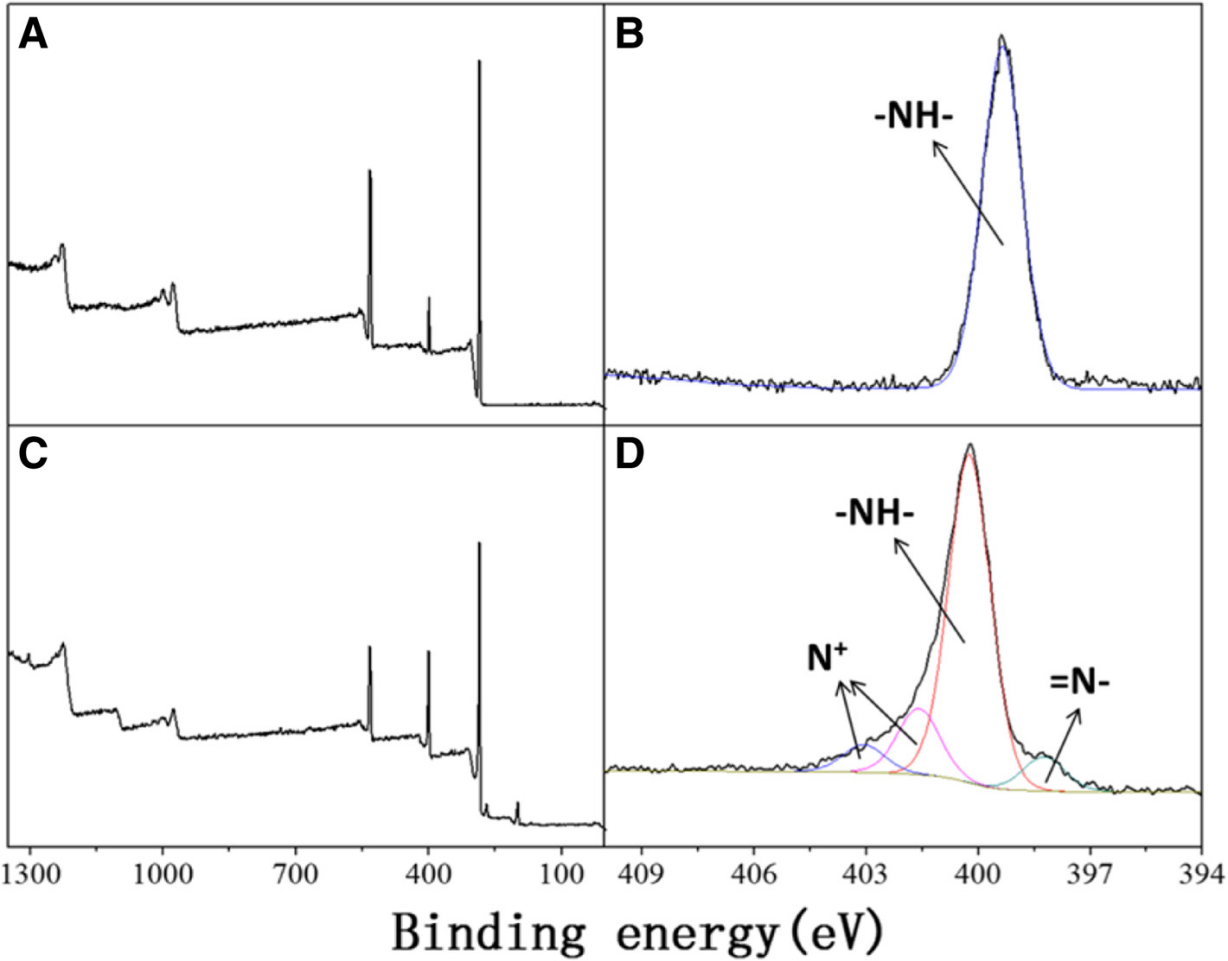
XPS wide scan and N 1 s regions of PA6 NFM **(A,B)**, PA6/PPy NFM **(C,D)**. The surface chemical compositions of PA6 nanofibers and PA6/PPy NFM were detected with X-ray photoelectron spectroscopy (XPS; ESCALAB 250Xi). The high-resolution N 1 s region of PA6 NFM showed there is an intense peak due to neutral amine nitrogen (−NH-) from the PA6 chains. When the pyrrole unit is doped, the main component of N 1 s peak shifts corresponding to the pyrrole nitrogen (−NH-). Besides, the peaks of imine nitrogen (=N-) and positively charged nitrogen atoms (N^+^) appears.

The XPS analysis results (Figure 3) give some insight into the chemical composition of the PA6/PPy NFM. The wide scans of PA6 and PA6 NFM were shown in Figure 3A,C. In the high-resolution N 1 s region of PA6 nanofibers (Figure 3B), there is an intense peak at 399.23 eV due to neutral amine nitrogen (−NH-) from the PA6 chains. When the pyrrole unit is doped (Figure 3D), the main component of N 1 s peak shifts to 400.23 eV corresponding to the pyrrole nitrogen (−NH-) [32]. At the same time, the minor components at low (398.23 eV) and high (401.63, 403.08 eV) binding energies are assigned to imine nitrogen (=N-) and positively charged nitrogen atoms (N^+^), respectively [33]. All the results above indicate that PPy layers have completely coated on the PA6 nanofibers.

### Adsorption mechanism study

#### π-π interactions

Two different concentrations were used to compare the adsorption capacities of PA6 and PA6/PPy NFM on atrazine. The equilibrium adsorption capacity (*q*_e_) of PA6 and PPy/PA6 NFM was 0.144 and 0.33 mg/g for 1 mg/L. While at 10 mg/L, *q*_e_ was increased to 0.55 and 3.2 mg/g for the two nanofibers mat. The *q*_e_ of PA6/PPy NFM was significantly higher than that of PA6 NFM at both initial concentrations tested, with 9.6× difference between the two at the high concentration of 10 mg/L. Although when the initial concentrations increased, the *q*_e_ of PA6 NFM and PA6/PPy NFM was both increased, it should be noticed that the *q*_e_ of PA6/PPy NFM at 10 mg/L was nearly ten times higher than that at 1 mg/L, while for PA6 NFM, the *q*_e_ only increased three times with the same concentration increased. That is to say, when PA6 nanofibers were functionalized with PPy, PA6/PPy NFM exhibited a significant increase adsorption capacity.

Compared with PA6 NFM, the rougher surface of PA6/PPy NFM could supply more adsorption sites due to its higher specific surface area, which resulted in a higher *q*_e_. Indeed, the surface area of PA6/PPy NFM was 32.5 m^2^/g, and for PA6 NFM, a lower surface area of 21.9 m^2^/g was obtained. However, the minor difference in surface areas could not convincingly explain the notably higher *q*_e_ of atrazine adsorbed onto PA6/PPy NFM, especially at the concentration of 10 mg/L. It indicated that there were more reasonable causations during the adsorption process.

**Figure 4:**
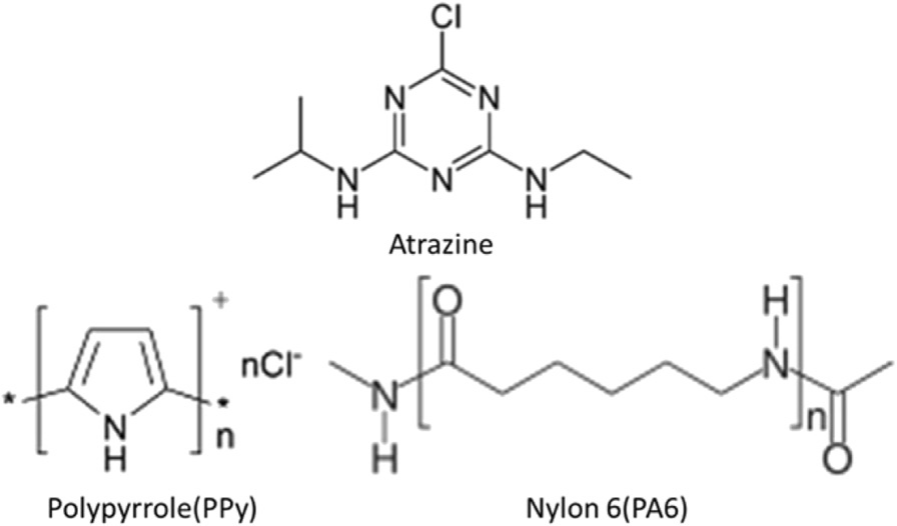
The molecular structure of atrazine, PPy, and PA6.

The molecular structure in Figure 4 has shown that the aromatic ring of PPy enabled π-π interaction with triazine ring of atrazine. But for PA6, without unsaturated bonds in its molecular skeleton, it could not adsorb atrazine through π-π interactions, which resulted in distinctively lower *q*_e_ of atrazine adsorbed onto PA6 NFM. It indicated that π-π interactions may be a major mechanism of atrazine adsorption onto the PA6/PPy NFM.

#### Electrostatic interaction

There is a significant effect of solution pH on the uptake of atrazine, since it impacts the surface charge of the adsorbent and the degree of ionization of the adsorbates [8]. When the pH of the initial solution changed from 3 to 11, the *q*_e_ was increased from 1.36 to 2.57 mg/g.

The Equations 3 and 4 show that PPy can be positively or negatively charged when solution pH is lower or higher than its isoelectric point (pI), where X is the counter anion. The pI of PPy prepared with Cl^−^ as the dopant was probably 10 [34]. The result of the Zeta electric potential also proved that PPy can be positively charged in a solution with a solution pH lower than 10 [35]:

3 $$ \mathrm{PPyX}+{\mathrm{H}}^{+}\leftrightarrow {\mathrm{PPyXH}}^{+},\kern0.62em \mathrm{p}\mathrm{H}<\mathrm{pI} $$

4 $$ \mathrm{PPyX}+{\mathrm{OH}}^{-}\leftrightarrow {\mathrm{PPyXOH}}^{-},\kern0.62em \mathrm{p}\mathrm{H}>\mathrm{pI} $$

Under the acid condition, atrazine was positively charged due to protonation, leading to stronger electrostatic repulsive interactions with the positively charged PPy, which therefore resulted in lower *q*_e_. However, the degree of atrazine protonation may decrease with the increase of pH, which can weaken the electrostatic repulsion. When the pH is higher than the pI of PPy (pH 10), the electrostatic repulsion can turn into electrostatic attraction due to the negative charges on PPy, which contributes to higher extraction efficiency, although the positive charges on atrazine were declined with the decrease of protonation. Hence, the presence of electrostatic interaction between atrazine and PA6/PPy NFM during the adsorption process was revealed.

It is further demonstrated from the results of the effect of ionic strength that the electrostatic interaction between atrazine and PA6/PPy NFM might be the possible mechanism during the adsorption process. The *q*_e_ increases to 49% with the 10% increase of NaCl concentration (from 2.18 to 3.25 mg/g) at pH 7, similarly.

Electrical double layers can form near PPy NFM surface contacting with the solution. At pH 7, PPy is positively charged, so anions in the solution can be attracted to the interface, forming the stern layer, while the adsorbate molecules are dispersed in the diffuse layer. The electrical double layers that are composed of stern and diffuse layers have a certain thickness, which can block the approach of the adsorption for the adsorbate to the adsorbents. But when the ionic strength of the solutions increases, the electrolyte (e.g., NaCl) will compress the electrical double layers and then weaken the electrostatic interaction between the adsorbates and adsorbents [36]. Since the electrostatic interaction between PPy and atrazine is a repulsive force at pH 7 as mentioned above, the weakened electrostatic interaction is beneficial to the adsorption process.

In conclusion, π-π interactions and electrostatic interactions were the main interaction forces for adsorption of atrazine on PA6/PPy NFM, with the two acting together as a mixed mode.

**Figure 5:**
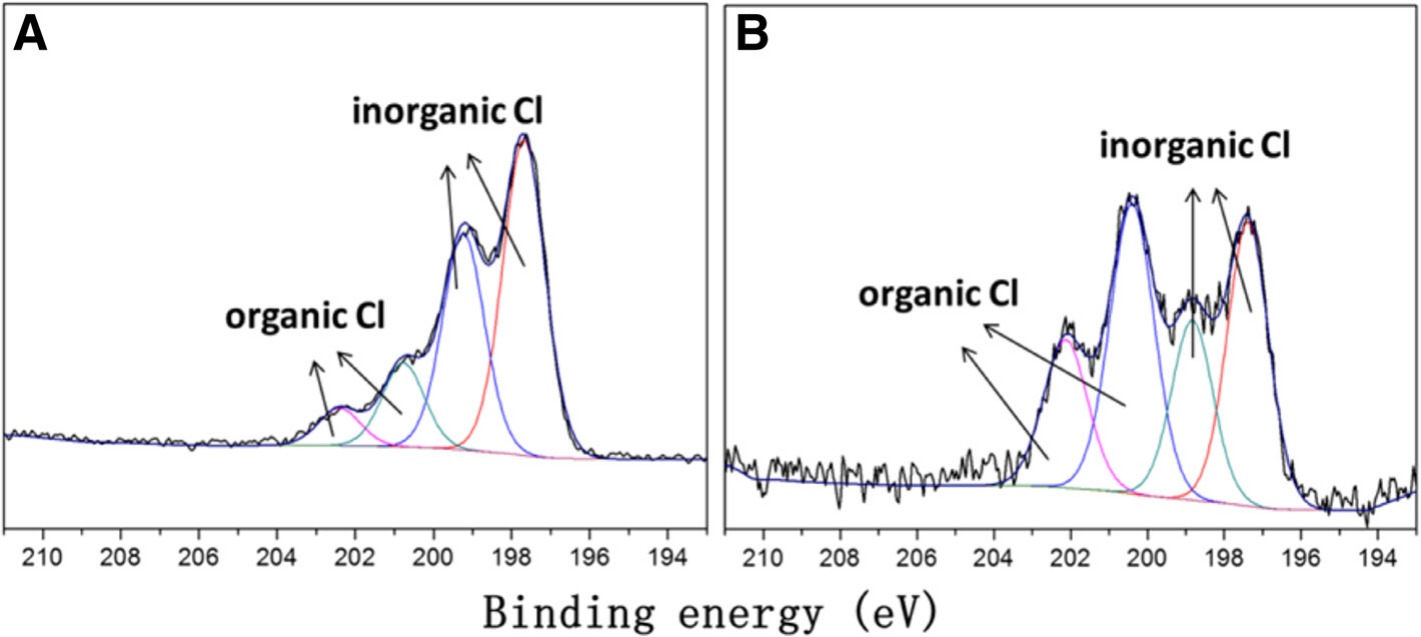
XPS Cl 2p core level spectra of PA6/PPy NFM before **(A)** and after **(B)** adsorption. The surface chemical compositions of PA6 nanofibers and PA6/PPy NFM were detected with XPS (ESCALAB 250Xi). The two spectra exhibited as four main peak. The four main peaks can be divided into two parts: inorganic chlorine and organic chlorine. An increase in the ratio of organic Cl with inorganic Cl was observed after adsorption.

**Figure 6:**
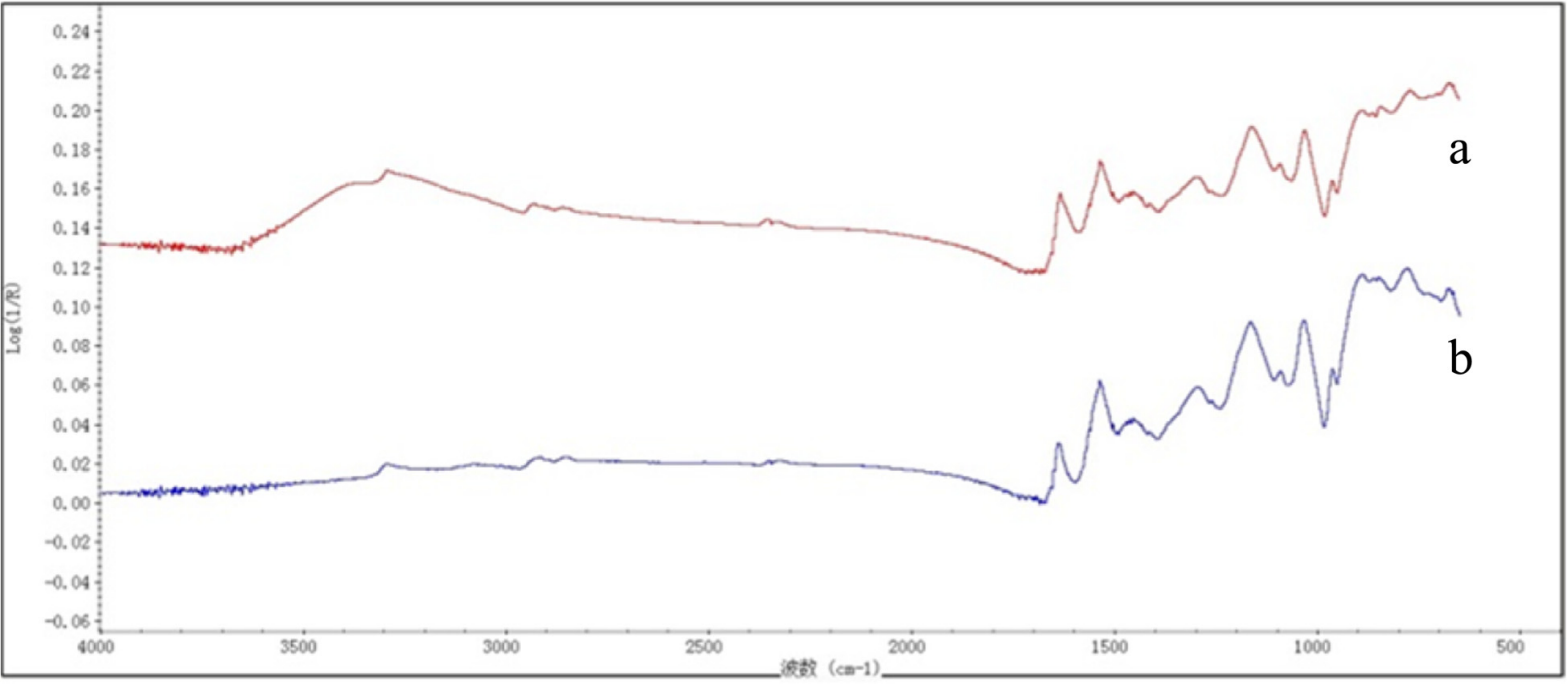
ATR-FTIR spectra of PA6/PPy NFM before **(a)** and after **(b)** adsorption. Fourier transform infrared (FTIR) spectra were carried out on a FTIR spectrophotometer (NEXUS 870). In the ATR-FTIR spectra of PA6/PPy NFM before and after adsorption, there was also no significant difference, which implies that no new species generated after adsorption.

Figure 5A,B presents the Cl 2p XPS spectra of PA6/PPy NFM before and after adsorption. The two spectra observed, shown by the black line, were broad in the binding energy range of 195.5 to 204.5 eV and exhibited the four main peaks at 197.5, 199, 200.5, and 202.2 eV approximately. Cl (2p) with a binding energy of <199 eV can unambiguously be assigned to inorganic chlorine, and Cl (2p) values of >200 eV characterize organic chlorine bonds (C-Cl) [37]. It can therefore divide the four main peaks into two parts: inorganic chlorine (the two peaks with lower BEs) and organic chlorine (the two peaks with higher BEs). An increase in the ratio of organic Cl with inorganic Cl (from 0.295 to 1.006) was observed after adsorption. It could be inferred that this effect is purely caused by the C-Cl bond in atrazine (Figure 4). The rise in ratio of C-Cl bond directly proved that the PA6/PPy NFM has successfully adsorbed atrazine. While in the attenuated total reflectance-Fourier transform infrared spectroscopy (ATR-FTIR) spectra of PA6/PPy NFM before and after adsorption, there was also no difference between Figure 6a and b, which implies that no new species was generated after adsorption. The adsorption of atrazine on PA6/PPy NFM was a physical adsorption, according with the assumption of π-π interactions and electrostatic interactions. The two forces dominated the adsorption process, with both of them acting as a mixed mode together, leading to the notable increase of the adsorption capacity.

### Adsorption isotherm and thermodynamic study

#### Adsorption kinetics

**Figure 7:**
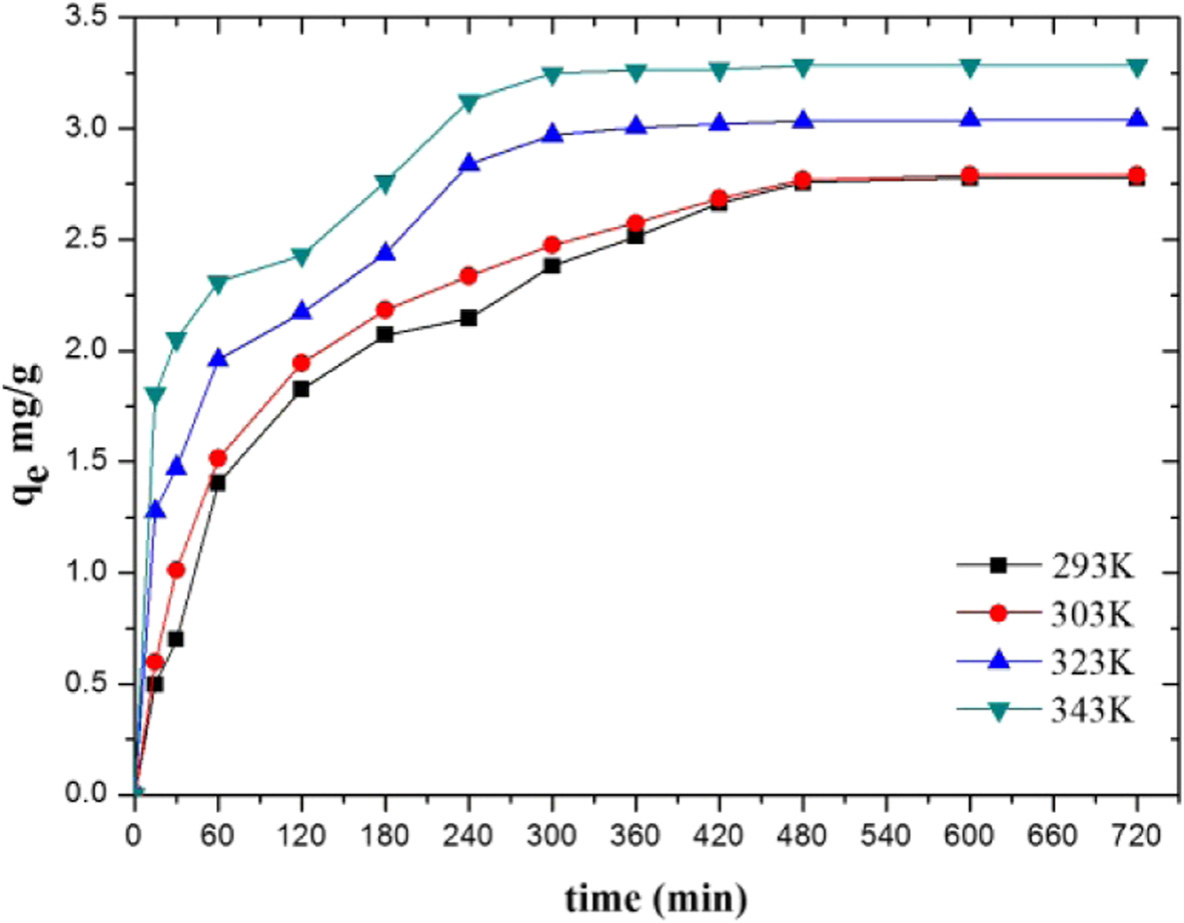
Effect of contact time on the adsorption of atrazine at different temperature. A 2.0-mg PA6/PPy NFM was immersed into 2 mL aqueous solution at 10 mg/L at 293, 303, 323, and 343 K; 50 μL samples withdrawn from the solutions were collected at time intervals of 5, 10, 15, 30,60,120,180,240,300,360,420,480,600,720 min for atrazine analysis.

The effect of adsorption time on the adsorption capacity at different temperatures is shown in Figure 7. The equilibrium time decreased from 480 to 300 min as the solution temperature increased from 293 to 343 K. The results clearly showed that the *q*_e_ of atrazine increased with the increase of temperature, which suggested that the process was endothermic [38]. The *q*_e_ was 2.77, 2.78, 3.04, and 3.28 mg/g for 293, 303, 323, and 343 K, respectively.

In order to better understand the adsorption process, the adsorption kinetics was investigated using the pseudo-first-order and pseudo-second-order models, according to the following Equations 5 and 6 respectively [18]:

5 $$ 1\mathrm{g}\left({\mathrm{q}}_{\mathrm{e}\hbox{-} }{\mathrm{q}}_{\mathrm{t}}\right)=1{\mathrm{gq}}_{\mathrm{e}}\hbox{-} \frac{{\mathrm{K}}_1}{2.303}\mathrm{t} $$

where *qt* is the adsorption capacity at time *t* (mg/g), *q*_e_ is the equilibrium adsorption capacity (mg/g), and *K*_1_ is the pseudo-first-order rate constants (1/min).

The plot of lg(*q*_e_ − *qt*) *versus t* gives a straight line with a slope of − *K*_1_ and an intercept of lg*q*_e_ [18]:

6 $$ \frac{t}{{\mathrm{q}}_{\mathrm{t}}}=\frac{1}{{\mathrm{K}}_2{{\mathrm{q}}_{\mathrm{e}}}^2}+\frac{1}{{\mathrm{q}}_{\mathrm{e}}}t $$

where *q*_*t*_ is the adsorption capacity at time *t* (mg/g), *q*_e_ is the equilibrium adsorption capacity (mg/g), and *K*_2_ is the pseudo-second-order rate constants (g/mg/min).

The plot of *t*/*q*_*t*_*versus t* gives a straight line with a slope of 1/*q*_e_ and an intercept of 1/(*K*_2_*q*_e_^2^).

**Figure 8:**
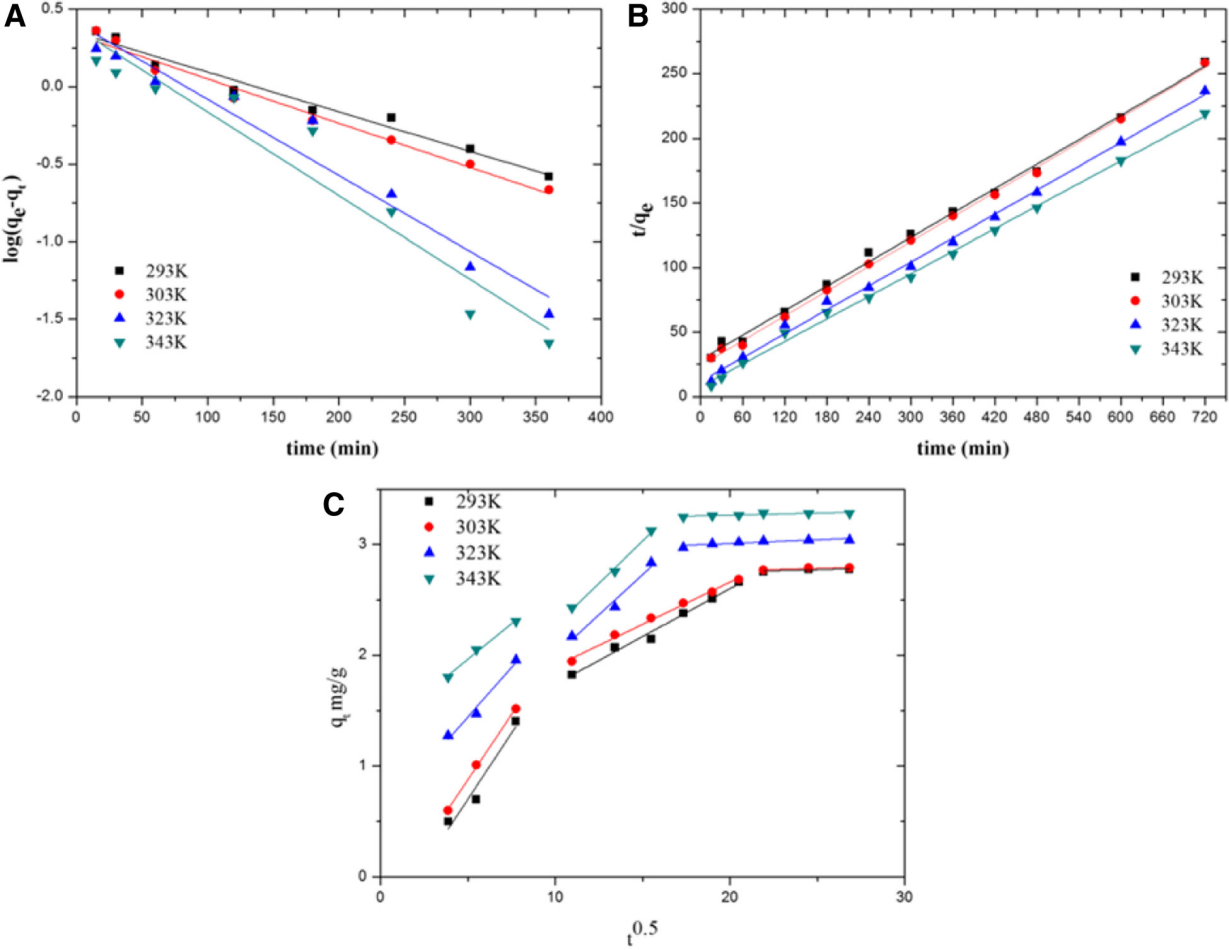
Pseudo-first-order kinetic model **(A)**, pseudo-second-order kinetic model **(B)** and intra-particle diffusion model **(C)** for the adsorption. The adsorption kinetic was investigated using the pseudo-first-order and pseudo-second-order models. The Weber-Morris intra-particle diffusion model was used to determine if intra-particle diffusion is the rate-limiting step.

The adsorption kinetic plots of atrazine are displayed in Figure 8A,B. From the plots, the related parameters were calculated and listed in Table 1. According to the values of the correlation coefficients (*R*^2^) at different temperatures, the pseudo-second-order model (*R*^2^ > 0.99) gave better description of atrazine adsorption on PA6/PPy NFM compared with the pseudo-first-order model (*R*^2^ > 0.92). The pseudo-second-order rate constant (*K*_2_) increased from 0.0034 to 0.0108 g/mg/min with temperature increased from 293 to 343 K. Furthermore, the *q*_e_ calculated by the pseudo-second-order model was closer to the values that were experimentally observed. The results accordingly indicated that the adsorption kinetics of atrazine adsorbed onto the PA6/PPy NFM was closer to the pseudo-second-order kinetic model than the pseudo-first-order kinetic model, suggesting that the adsorption process was a multi-stage process.

**Table 1:** **Adsorption kinetic model rate constants for atrazine adsorption on PA6/PPy NFM at different temperatures**

**Temperature (K)**	***q***_**e**_ **,**_**exp**_ **(mg/g)**	**Pseudo-first-order model**			**Pseudo-second-order model**		
		***K***_**1**_ **(1/min)**	***q***_**e,cal**_ **(mg/g)**	***R***_**1**_^**2**^	***K***_**2**_ **(g/mg/min)**	***q***_**e,cal**_ **(mg/g)**	***R***_**2**_^**2**^
293	2.77	0.0059	2.22	0.972	0.0034	3.12	0.996
303	2.78	0.0066	2.15	0.984	0.0042	3.17	0.999
323	3.04	0.0113	2.56	0.949	0.0081	3.24	0.997
343	3.28	0.0124	2.38	0.921	0.0108	3.43	0.998

To our knowledge, there is no relevant model to predict mat or membrane diffusion. The Weber-Morris intra-particle diffusion model has often been used to determine if intra-particle diffusion is the rate-limiting step [39]. According to this model, the adsorption process for nanofibers mat can be divided into three stages, including the diffusion from the bulk solution to the external surface of nanofibers mat (called ‘external diffusion’), pore diffusion inside the nanofibers mat (called ‘intra-mat diffusion’), and the ‘internal diffusion’ to the adsorption sites. Here, we established the ‘intra-mat diffusion model’ and identified the rate-controlling step in order to more clearly describe the adsorption process. The model was represented by the following Equation 7 [38]:

7 $$ {q}_t={K}_m{t}^{1/2}+C $$

where *q*_*t*_ is the adsorption capacity at time *t* (mg/g), *K*_*m*_ is the adsorption rate constants of intra-mat diffusion model (mg/g/min^1/2^) and *C* is the intercept which is related to the thickness of the boundary layer.

The plot of *q*_*t*_*versus t*^1/2^ gives a straight line with a slope of *K*_*m*_ and an intercept of *C*.

Based on this model, the plot should be linear if the intra-mat diffusion is involved in the adsorption process and the plot passes through the origin then intra-mat diffusion is the sole rate-controlling step [19]. As shown in Figure 8C, the plot did not pass through the origin; instead, three linear portions were obtained, which suggested that the adsorption occurred in all three phases/stages including the external diffusion, intra-mat diffusion, and the internal diffusion stage. These results indicated that intra-mat diffusion was not the only rate-limiting step. It may also suggest that the external diffusion assisted by agitation or oscillation can benefit the adsorption process, while our work was completed under static adsorption conditions where adsorption equilibrium was established by simple diffusion.

#### Adsorption isotherm

To study the interaction between atrazine and PA6/PPy NFM, two well-known models of Langmuir and Freundlich isotherms were used to fit the equilibrium data.

The linearized Langmuir isotherm model is represented by the following equation [18]:

8 $$ \frac{C_{\mathrm{e}}}{{\mathrm{q}}_{\mathrm{e}}}=\frac{C_{\mathrm{e}}}{{\mathrm{q}}_{max}}+\frac{1}{{\mathrm{K}}_{\mathrm{L}}{\mathrm{q}}_{max}} $$

where *C*_e_ is the equilibrium concentration (mg/L), *q*_e_ is the equilibrium adsorption capacity (mg/g), *q*_max_ is the maximum adsorption capacity (mg/g), and *K*_L_ is the adsorption equilibrium constant of Langmuir model (L/mg).

Equation 8 implies that a plot of *C*_e_/*q*_e_ against *C*e should be a straight line, with *q*_max_ and *K*_L_ being calculated.

The linearized Freundlich isotherm model is represented by the following equation [18]:

9 $$ 1\mathrm{g}{q}_e=1\mathrm{g}{K}_{\mathrm{F}}+\frac{1}{n}1\mathrm{g}{C}_e $$

where *C*_e_ is the equilibrium concentration (mg/L), *q*_e_ is the equilibrium adsorption capacity (mg/g), *K*_F_ and 1/*n* are Freundlich model constants that are related to the adsorption capacity.

Equation 9 implies that a plot of lg*q*_e_ against lg*C*_e_ should be a straight line, with *K*_F_ and *n* calculated.

The linear regression results of the two isotherm models are shown in Figure 8A,B. In addition, the detailed experimental parameters calculated from Langmuir and Freundlich isotherm models are summarized in Table 2. It is obvious that the adsorption behaviors of atrazine onto PA6/PPy NFM fitted better with the Freundlich isotherm with higher *R*^2^ at each temperature than that of Langmuir model. From the calculated results of *Q*_max_ in the Langmuir model, it decreased with the rise in temperature, which was contradicted with the above conclusion of endothermic process. It was another evidence that the Langmuir model could not describe the adsorption behavior more perfectly than the Freundlich model.

**Table 2:** **Langmuir and Freundlich constants for atrazine adsorption on PA6/PPy NFM**

***Temperature (K)***	***Langmuir***			***Freundlich***		
	***K***_***L***_	***q***_***max***_ ***(mg/g)***	***R***_***1***_^***2***^	***K***_***F***_	***n***	***R***_***2***_^***2***^
293	0.40	2.74	0.963	0.76	1.49	0.999
323	0.94	2.16	0.929	2.04	2.03	0.987
343	2.55	2.04	0.971	2.45	2.46	0.990

The Langmuir model is based on the assumption of adsorption homogeneity, supposing equal availability of adsorption sites, monolayer surface coverage, and no interaction between adsorbed species [40]. The limitation of the model also leads to greater error between calculated *q*_max_ and experimental results. In contrast, the Freundlich model describes reversible adsorption and is not restricted to the formation of the monolayer. The value of *n* was greater than 1, which indicated that the adsorption process is favored [39]. The rise in the value of *n* with temperature also indicated that the adsorption reaction is an endothermic process.

#### Adsorption thermodynamics

Gibbs equation is utilized to study the temperature effect on equilibrium adsorption. The equation can be expressed as follows [18]:

10 $$ \Delta \mathrm{G}{}^{\circ}=-RT \ln {K}_{\mathrm{d}} $$

where ΔG° is standard free energy (kJ/mol), *R* is the universal gas constant (8.314 J/mol K), *T* is the absolute solution temperature (K), and *K*_d_ is distribution adsorption coefficient which can be determined by the following equation [18]:

11 $$ {K}_{\mathrm{d}}=\frac{C_{\mathrm{o}}\hbox{-} {C}_{\mathrm{e}}}{C_{\mathrm{e}}}\kern0.24em \frac{V}{m} $$

where *C*_0_ is the initial concentration (mg/L), *C*_e_ is the equilibration concentration after adsorption (mg/L), *V* is the volume of the solution (L), and *m* is the dose of the nanofiber mat (g).

Gibbs equation can also be explained as follows [18]:

12 $$ \Delta \mathrm{G}{}^{\circ}=\Delta \mathrm{H}{}^{\circ}-T\Delta \mathrm{S}{}^{\circ} $$

where ΔG° is standard free energy (kJ/mol), ΔH° is standard enthalpy (kJ/mol), ΔS° is standard entropy (J/mol/K), and *T* is the absolute solution temperature (K).

Combining above equations will give us Equation 13 [18]:

13 $$ 1\mathrm{n}{K}_{\mathrm{d}}=\frac{{\Delta \mathrm{S}}^0}{R}-\frac{{\Delta \mathrm{H}}^0}{RT} $$

**Figure 9:**
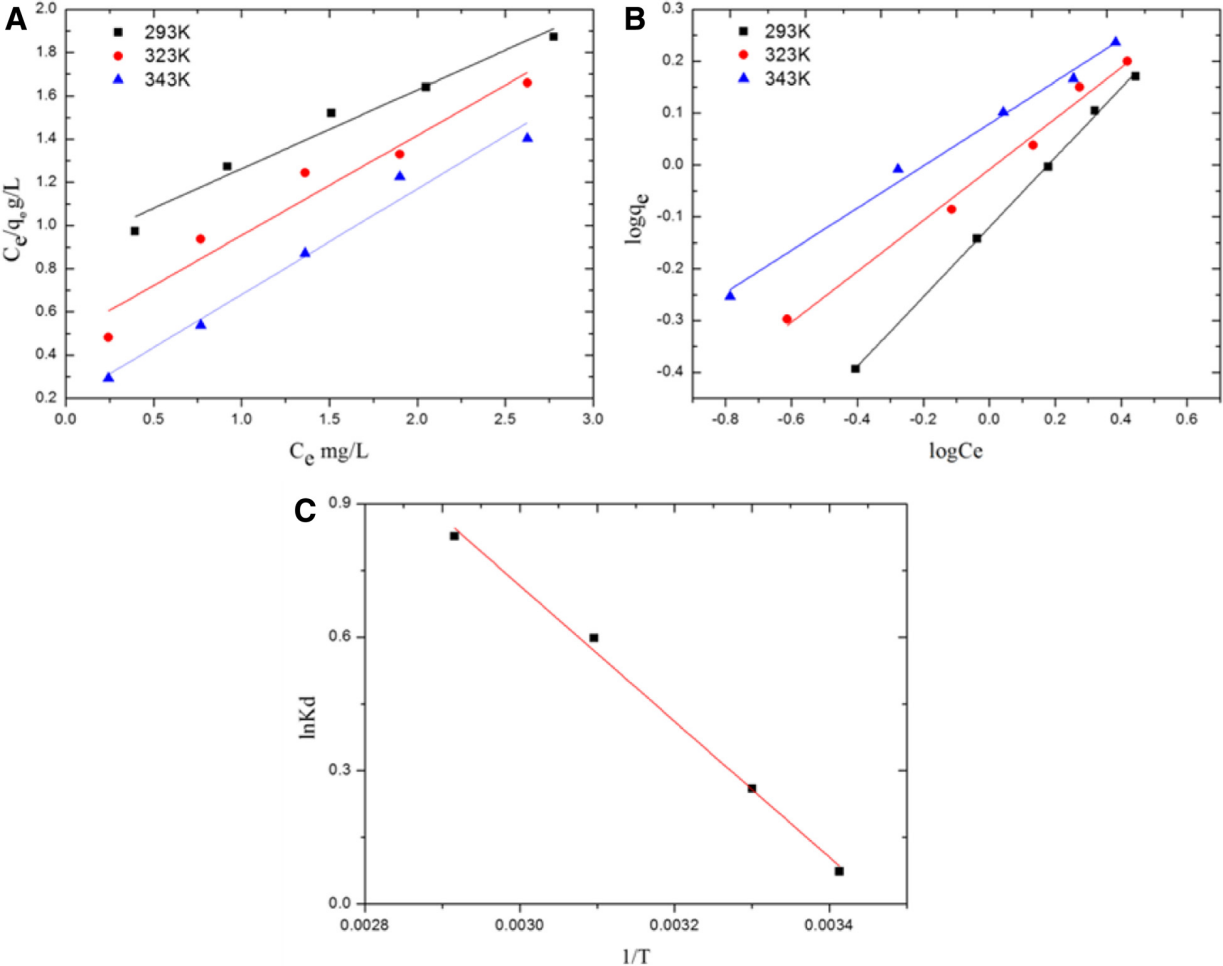
Langmuir **(A)** and Freundlich **(B)** isotherms model for adsorption. Plot to estimate thermodynamic parameters for adsorption **(C)**. Two well-known models of Langmuir and Freundlich isotherms were used to fit the equilibrium data. The plot of ln*K*_d_ versus 1/*T* gives a straight line with a slope of ΔH° and an intercept of ΔS°.

As shown in Figure 9C, the plot of ln*K*_d_*versus* 1/*T* gives a straight line with a slope of ΔH° and an intercept of ΔS°. The values of atrazine parameters (measured at different temperatures) are presented in Table 3.

**Table 3:** **Adsorption thermodynamics parameters for atrazine adsorption on PA6/PPy NFM**

**Temperature (K)**	**ΔG° (kJ/mol)**	**ΔH° (kJ/mol)**	**ΔS° (kJ/mol/K)**
293	−0.177	12.7	0.044
303	−0.653		
323	−1.606		
343	−2.356		

From the listed data in Table 3, the adsorption of atrazine on PA6/PPy NFM was a spontaneous (ΔG° < 0) and endothermic (ΔH° > 0) process. The value of ΔG° was in the range of 0 to −20 kJ/mol, falling in the standard free energy range of physical adsorption, which indicated that it was a physical adsorption of atrazine [41]. The positive values of ΔS° suggested the increase in randomness at the solid/solution interface during the adsorption of atrazine in aqueous solution on the PA6/PPy NFM. The decrease in ΔG° values with an increase in temperature indicated that heating will promote the adsorption.

#### Maximum adsorption capacity

In order to investigate the maximum adsorption capacity of PA6/PPy NFM on atrazine, different initial concentrations (*C*_0_) from 1 to 200 mg/L were used to accomplish the experiment. The *q*_e_ increased with initial concentration, especially during the beginning phase. This suggested that the rate of external diffusion accelerated with the initial concentration, resulting in more target molecules absorbing in the aperture of nanofibers mat. When the initial concentration increased to 120 mg/L, the adsorption sites were close to saturation, with the curve becoming shallower. The maximum adsorption capacity of 14.8 mg/g was reached at the concentration of 200 mg/L.

### Reusability of PA6/PPy NFM

During the fiber reusability study, no significant change of the *q*_e_ was observed after five usages. There is a decrease of only 0.2 mg/g in the *q*_e_ after six usages, which is almost remained unchanged based on statistical analysis (*P* > 0.05). The excellent reusability of the nanofibers mat will save cost for atrazine removal thus have huge economic benefits.

### Comparison of atrazine adsorption between the PA6/PPy NFM and other adsorbent materials

Table 4 listed the adsorption performance of other sorbent materials reported in literature. Activated carbon/iron oxide composites seemed to have the best adsorption capability. The possible reason was that the oxidation of iron oxide played an important role during the adsorption process, in addition to the function of activated carbon [42]. However, the byproducts of iron oxidation and the desorption difficulty of activated carbon limited the practical uses. Here we have demonstrated that the adsorption capacity of PA6/PPy NFM was superior to other existing adsorbent materials. The advantages of high desorption ability and no need for secondary separation make the PA6/PPy NFM more convenient in practical use.

**Table 4:** **Comparison with other sorbent materials in literatures**

**Type of adsorbent**	**Adsorbent dose**	**Maximum adsorption capacity**	**Reference**
Activated carbon/iron oxide composites	10 mg	30 mg/g	[42]
Diatomaceous earth	10 mg	1.1 mg/g	[43]
Organo-zeolite	5 g	0.43 mg/g	[44]
Granular activated carbon	100 mg	7.5 mg/g	[45]
Nanoscale zero valent iron	200 mg	8.89 mg/g	[46]
PA6/PPy NFM	2.0 mg	14.8 mg/g	This study

## Conclusions

In this study, PA6/PPy NFM was used as an adsorbent to remove atrazine from aqueous solution, in an attempt to explore the feasibility of using nanofibers-based adsorption technique for the removal of atrazine in contaminated water. Key experiments were completed to elucidate the detailed adsorption characteristics and the possible mechanism. According to our results, PA6/PPy NFM has the great potential to be a new atrazine adsorbent for wastewater treatment due to its significantly higher removal efficiency than that of most existing atrazine removal sorbents published in literature. Further study is in progress to verify the adsorption performance of PA6/PPy NFM in the removal of atrazine from contaminated water.
